# Novel, clinically relevant genomic patterns identified by comprehensive genomic profiling in ATRX-deficient IDH-wildtype adult high-grade gliomas

**DOI:** 10.1038/s41598-023-45786-w

**Published:** 2023-10-27

**Authors:** Gábor Bedics, Péter Szőke, Bence Bátai, Tibor Nagy, Gergő Papp, Noémi Kránitz, Hajnalka Rajnai, Lilla Reiniger, Csaba Bödör, Bálint Scheich

**Affiliations:** 1 Department of Pathology and Experimental Cancer Research, Semmelweis University, Üllői út 26, Budapest, 1085 Hungary; 2 HCEMM-SE Molecular Oncohematology Research Group, Department of Pathology and Experimental Cancer Research, Semmelweis University, Üllői út 26, Budapest, 1085 Hungary; 3 Department of Biochemistry and Molecular Biology, Faculty of Medicine, University of Debrecen, Egyetem tér 1, Life Science Building, Debrecen, 4032 Hungary; 4 Department of Pathology, County Hospital Győr, Petz Aladár Hospital, Vasvári Pál út 2-4, Győr, 9024 Hungary

**Keywords:** Cancer, Neurological disorders, Cancer genetics, CNS cancer

## Abstract

Glioblastomas are the most common IDH-wildtype adult high-grade gliomas, frequently harboring mutations in the *TERT* gene promoter (*pTERT*) and utilizing the subsequent telomerase overexpression for telomere length maintenance. However, some rare cases show loss of ATRX and use alternative mechanisms of telomere lengthening. In this study, we performed the first complex genomic analysis specifically concentrating on the latter subgroup. Comprehensive genomic profiling of 12 ATRX-deficient and 13 ATRX-intact IDH-wildtype adult high-grade gliomas revealed that *ATRX* and *pTERT* mutations are mutually exclusive. *DNMT3A* alterations were confined to ATRX-deficient, while *PTEN* mutations to ATRX-intact cases. RAS–MAPK pathway alterations, including *NF1* mutations, were more characteristic in the ATRX-deficient group. Variants of genes related to homologous recombination repair showed different patterns of affected genes. Two ATRX-deficient tumors with high tumor mutational burden and mismatch repair deficiency were found. One of these contained a novel fusion involving the *NTRK2* and *LRRFIP2* genes, while the other showed loss of MSH2 and MSH6 without genetic alterations in the encoding genes suggesting an epigenetic background. Genetic characteristics of ATRX-deficient IDH-wildtype adult high-grade gliomas suggest that these tumors are particularly intriguing targets of potential future therapeutic interventions including immunotherapies combined with MAPK pathway inhibition and DNA repair inhibitors.

## Introduction

Glioblastoma [GB; World Health Organization (WHO) grade 4] is the most common adult-type high-grade glioma, currently defined as an IDH-wildtype and H3-wildtype diffuse astrocytic tumor^1^. The prognosis is still extremely poor and effective therapeutic interventions are lacking. Most molecularly targeted and immunotherapeutic approaches failed to show significant effects in clinical trials^2^. Therefore, detailed characterization of GB subgroups and the search for novel predictive biomarkers and actionable targets are crucial in order to develop future personalized treatment options.

Approximately 80% of GBs harbors hotspot mutations in the promoter region of the *TERT* gene (*pTERT*) currently considered as entity-defining molecular markers^3^. The subsequent overexpression of the encoded catalytic subunit of the telomerase enzyme is the main mechanism of telomere length maintenance in these tumors. In contrast, IDH-mutant astrocytomas and H3-altered high-grade pediatric-type diffuse gliomas are characterized by the loss of ATRX (α thalassemia/mental retardation syndrome X-linked), a chromatin-remodeling factor and histone chaperone^4^. Loss of ATRX protein induces alternative lengthening of telomeres (ALT), a telomerase-independent mechanism of telomere maintenance being dependent on homologous recombination (HR) between telomeric sequences^5^. Besides the abovementioned entities, ALT is also involved in the pathogenesis of a small subset of *pTERT*-wildtype GBs. This latter subgroup frequently harbors loss-of-function mutations of either *ATRX* or *SMARCAL1* genes as it was recently described^6^.

ATRX-deficient IDH-wildtype and H3-wildtype adult gliomas are rare and include mostly GBs and some recently described new entities, such as high-grade astrocytoma with piloid features (HGAP)^7^ and glioneuronal tumor with ATRX alteration, kinase fusion and anaplastic features (GTAKA)^8^. Many cases of these latter entities previously fell into the GB category. Due to the rarity of these tumors, only limited data are available regarding their genetic characteristics, particularly the clinically relevant molecular patterns. Therefore, further investigation is needed to elucidate their potential unique biological, genomic and clinical properties. In order to address this issue, we performed comprehensive genomic profiling (CGP) of an IDH-wildtype and H3-wildtype adult high-grade astrocytic glioma cohort showing ATRX loss (n = 12) in parallel with the analysis of an ATRX-intact GB cohort being similar in size (n = 13), as a clinically meaningful comparison. This was implemented using the Illumina TruSight Oncology 500 (TSO-500) targeted next-generation sequencing (NGS) panel. For the list of genes tested for small variants [single nucleotide variants (SNVs), small insertions and deletions (InDels), copy number variations (CNVs)] and fusions, see Supplementary Table S1.

## Results

### Clinical and histopathological characteristics of ATRX-deficient and ATRX-intact tumors

Twenty five adult IDH-wildtype and H3-wildtype gliomas showing obvious high-grade histological features were included in this study. Among these, 12 showed ATRX loss and 13 showed retained ATRX expression according to the immunohistochemical (IHC) analysis (Table 1). There was no significant difference in terms of patient age between the ATRX-deficient and ATRX-intact groups (*p* = 0.1241, Mann–Whitney test), but more patients above 60 years of age were included into the ATRX-deficient group (Supplementary Fig. S1a). Kaplan–Meier analysis did not reveal significant difference between the overall survival (OS) of ATRX-deficient and ATRX-intact cases (hazard ratio = 1.829, 95% confidence interval 0.7085–4.722, log-rank test: χ^2^ (1) = 1.98, *p* = 0.1593) (Supplementary Fig. S1b). However, median OS of the ATRX-deficient group is still conspicuously lower (Table 1) being probably related to the higher proportion of elderly patients and not to real biological difference. There are 3 cases with OS above 36 months and therefore considered to be long-term survivors^9^, 1 in the ATRX-deficient and 2 in the ATRX-intact groups. The majority of patients received radio-chemotherapy according to the standard guidelines which was complemented with bevacizumab in 13 cases. Oncological treatment could not be used in 3 cases due to the poor general status of the patients. Recurrent tumor samples were available for molecular analysis in 7 cases (Table 1). In these cases, only recurrent tumor samples were subjected to genetic testing.

**Table 1:** Clinico-pathological characteristics of the included cases in the ATRX-deficient and ATRX-intact groups.

	ATRX-deficient	ATRX-intact
*Total number of cases*	12 (100%)	13 (100%)
*Age—mean (range), years*	61.08 (34–87)	50.85 (36–63)
*Sex*		
Male	8 (66.7%)	9 (69.2%)
Female	4 (33.3%)	4 (30.8%)
*Localization*		
Supratentorial	10 (83.3%)	12 (92.3%)
Cerebellar	1 (8.3%)	1 (7.7%)
Spinal	1 (8.3%)	–
*Recurrent tumor*	2 (16.7%)	5 (38.5%)
*Median overall survival (months)*	5.03	14.00
*Diagnosis*		
High-grade astrocytoma with piloid features	1 (8.3%)	–
Astrocytoma, NEC (G3)	1 (8.3%)	–
Glioblastoma, IDH-wildtype (G4)	10 (83.3%)	13 (100%)
*Giant cells*		
Inconspicuous	5 (41.7%)	10 (76.9%)
< 30%	5 (41.7%)	2 (15.4%)
> 30%	2 (16.7%)	1 (7.7%)
*Therapy*		
No	3 (25%)	0 (0%)
Radiotherapy	9 (75%)	13 (100%)
Chemotherapy	7 (58.3%)	13 (100%)
Bevacizumab	1 (9.1%)	12 (92.3%)

In the ATRX-deficient group, 10 cases were diagnosed as GB (WHO grade 4) based on the morphological, IHC and available molecular data. One spinal tumor (case #2) in this group contained areas reminiscent of pilocytic astrocytoma and harbored *KIAA1549::BRAF* fusion (Supplementary Fig. S2b) together with ATRX loss being highly suggestive of HGAP^7^. Another tumor (case #3) was diagnosed as astrocytoma, not elsewhere classified (NEC) (WHO grade 3) from a stereotaxic biopsy sample due to the IDH-wildtype and H3-wildtype state and lack of criteria defining GB. In this case, the patient had received the clinical diagnosis of neurofibromatosis type I. Case #17 showed an oligodendroglioma-like morphology (see later), diffuse OLIG2 nuclear positivity, but only focal expression of GFAP. Fluorescent in situ hybridization (FISH) analysis revealed relative deletions of 1p36 and 19q13 chromosomal regions in 50% and 75% of the polysomic tumor cells, respectively. However, the IDH-wildtype state excluded the diagnosis of oligodendroglioma. These latter cases demonstrate some degree of heterogeneity of the ATRX-deficient group, but it is still in accordance with the originally defined group of interest. Each case in the ATRX-intact group was morphologically and molecularly consistent with GB (WHO grade 4). Readily identifiable multinucleated giant cells were present in 7 (58.3%) and 3 (23.1%) cases within the ATRX-deficient and ATRX-intact groups, respectively (*p* = 0.1107, Fisher’s exact test). However, giant cells were usually scattered and exceeded the proportion of 30% in only two ATRX-deficient and one ATRX-intact tumors.

### Genetic landscape of ATRX-deficient gliomas

**Figure 1:**
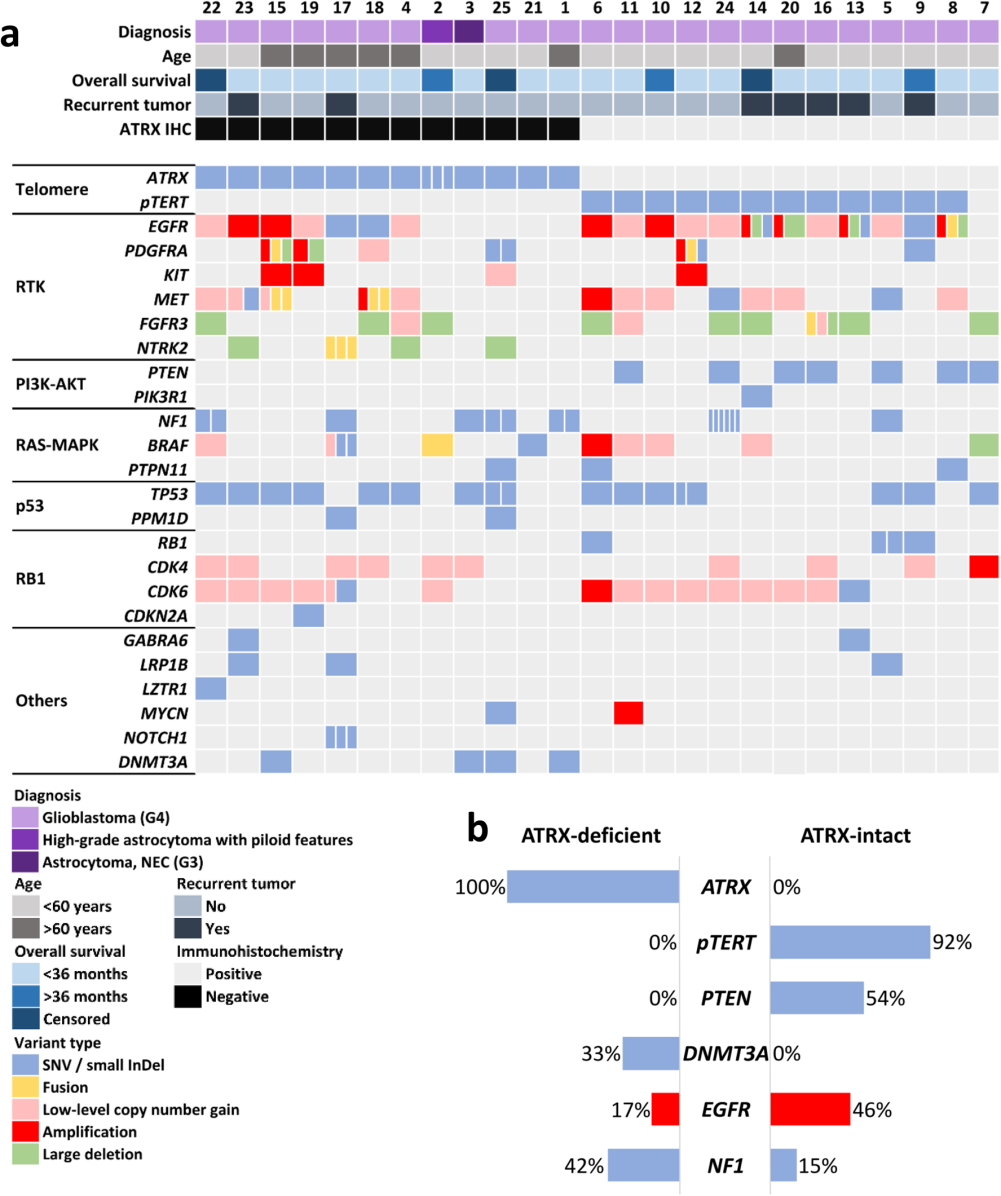
(**a**) Summary of the main clinical characteristics and alterations affecting genes well documented to be involved in GB pathogenesis in ATRX-deficient and ATRX-intact glioma cases (divided cells denote multiple variants in the same gene). (**b**) Comparison of *ATRX*, *pTERT*, *PTEN*, *DNMT3A* and *NF1* mutational as well as *EGFR* amplification frequencies in the ATRX-deficient and ATRX-intact groups, respectively.

NGS analysis revealed pathogenic (P) or likely pathogenic (LP) variants of *ATRX* in all ATRX-deficient, but in none of ATRX-intact tumors. *pTERT* mutations (namely c.-124C > T) were detected in 12 of 13 ATRX-intact, but none of ATRX-deficient gliomas (Fig. 1a,b). Mutual exclusivity of *ATRX* and *pTERT* mutations were confirmed by the statistical analysis (*p* = 2.692e−6; *p*_adj_ = 1.345e−5, Fisher’s exact test).

As expected, the most frequently affected gene among receptor tyrosine kinases (RTK) was *EGFR*. The occurrence of *EGFR* amplification was apparently, but not significantly lower in ATRX-deficient cases (*p* = 0.2016, *p*_adj_ = 0.2016, Fisher’s exact test) (Fig. 1a,b). Other RTKs, namely *PDGFRA*, *KIT* and *MET* were affected less frequently, with no obvious differences between the analyzed groups. Amplification of the *PDGFRA* gene was detected in 3 cases and accompanied by complex alterations including fusions, mutations and deletions as well as concurrent amplification of the *KIT* gene (Fig. 1a). Fusions of RTK genes were detected in 3 ATRX-deficient and 3 ATRX-intact cases, respectively (Supplementary Fig. S2a)^10^. These rearrangements included the *SEPTIN14::EGFR* (case #8), *PDGFRA::ADGRL3* (case #12), *KDR::PDGFRA*, *PTPRZ1::MET*, *WASL::MET* (case #15) and *DNAJB6::MET* (case #18) fusions. In addition, one GB (case #16) harboring *FGFR3::TACC3* fusion was found in the ATRX-intact group, but the previously reported characteristic morphological features^11^ frequently accompanying this fusion were not evident (Supplementary Fig. S2c). Notably, fusions affecting the *NTRK2* gene, namely *NTRK2::LRRFIP2* and *LRRFIP2::NTRK2* were detected in case #17 (Fig. 3i,j).

Among members of the PI3K-AKT pathway, *PTEN* was the most frequently affected gene in ATRX-intact tumors (53.9%). In contrast, none of the ATRX-deficient tumors harbored variants in *PTEN* fulfilling the inclusion criteria (*p* = 0.0052, *p*_adj_ = 0.013, Fisher’s exact test) (Fig. 1a,b). Regarding RAS–MAPK pathway, *NF1* mutations occurred in 42% of ATRX-deficient, while only 15% of ATRX-intact tumors, although the difference was not statistically significant (*p* = 0.2016, *p*_adj_ = 0.2016, Fisher’s exact test) (Fig. 1a, b). In addition, besides the one case harboring a *KIAA1549::BRAF* fusion, another contained *BRAF* p.V600E mutation in the ATRX-deficient group. *PTPN11* mutations occurred in 3 cases, among which the two P variants were detected in the ATRX-intact group. *TP53* mutations were frequent in both groups (66.7% and 53.9% in ATRX-deficient and ATRX-intact groups, respectively). *PPM1D*, an oncogene related to p53 signaling, was altered in 2 additional ATRX-deficient cases, namely the 2 cases characterized by high tumor mutational burden (TMB) (see later). Mutations of the *RB1* gene as well as *CDK4* and *CDK6* amplifications (but not low-level copy number gains) were found in scattered cases of the ATRX-intact group (Fig. 1a).

Among less frequently altered genes previously described to be affected in diffuse gliomas, *GABRA6*, *LRP1B*, *LZTR1*, *MYCN* and *NOTCH1* harbored P or LP mutations in scattered cases. Intriguingly, mutations of the *DNMT3A* gene were found in 4 cases, all of which were ATRX deficient (*p* = 0.0391, *p*_adj_ = 0.0652, Fisher’s exact test) (Fig. 1a, b).

**Figure 2:**
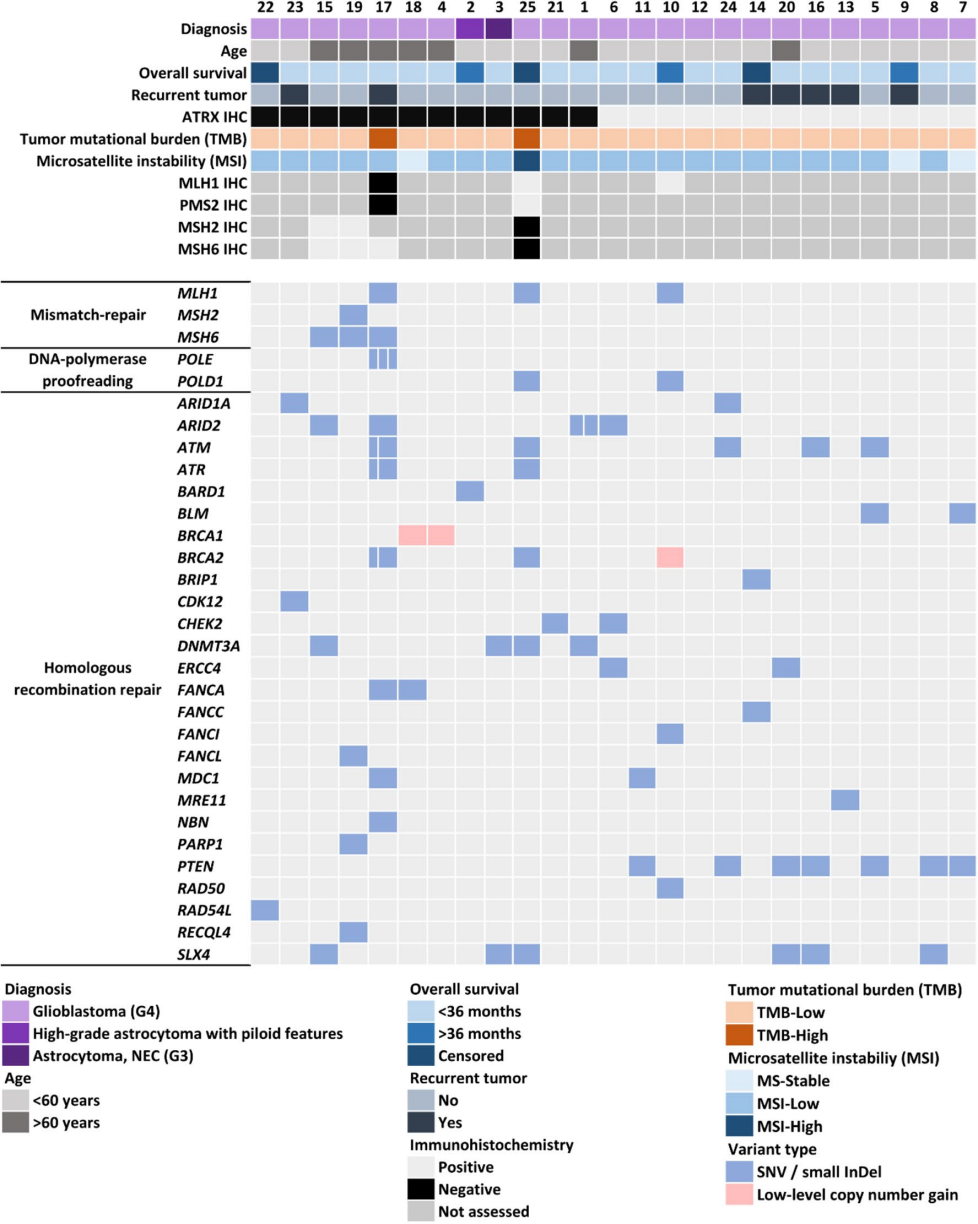
Summary of the main clinical characteristics and alterations affecting genes related to DNA repair, tumor mutational burden (TMB) and microsatellite instability (MSI) as well as results of mismatch repair protein immunohistochemical analyses in ATRX-deficient and ATRX-intact glioma cases (divided cells denote multiple variants in the same gene).

**Figure 3:**
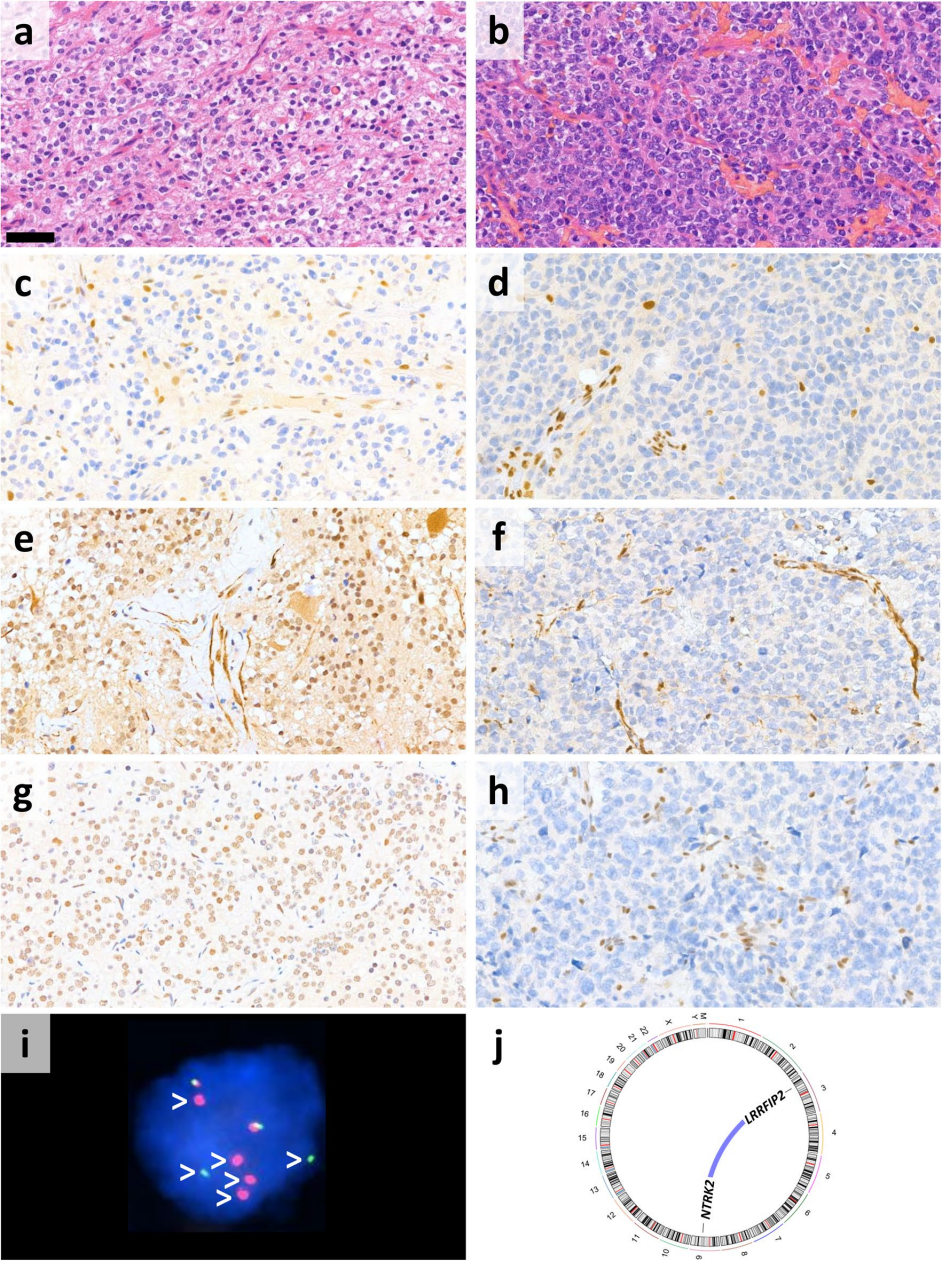
Oligodendroglioma-like morphology of case #17 shown by the H&E-stained sections from the (**a**) primary and (**b**) recurrent tumor resected following radio-chemotherapy (scale bar: 50 μm). (**c** and **d**) ATRX expression was lost in both samples. (**e**) MLH1 and (**g**) PMS2 were retained in the primary specimen, while both (**f**) MLH1 and (**h**) PMS2 were lost in the recurrent tumor. (**i**) *NTRK2* fluorescent in situ hybridization showed break apart signals (white arrowheads) in the tumor cells validating the presence of *NTRK2::LRRFIP2* and *LRRFIP2::NTRK2* fusions revealed by next-generation sequencing (**j**).

**Figure 4:**
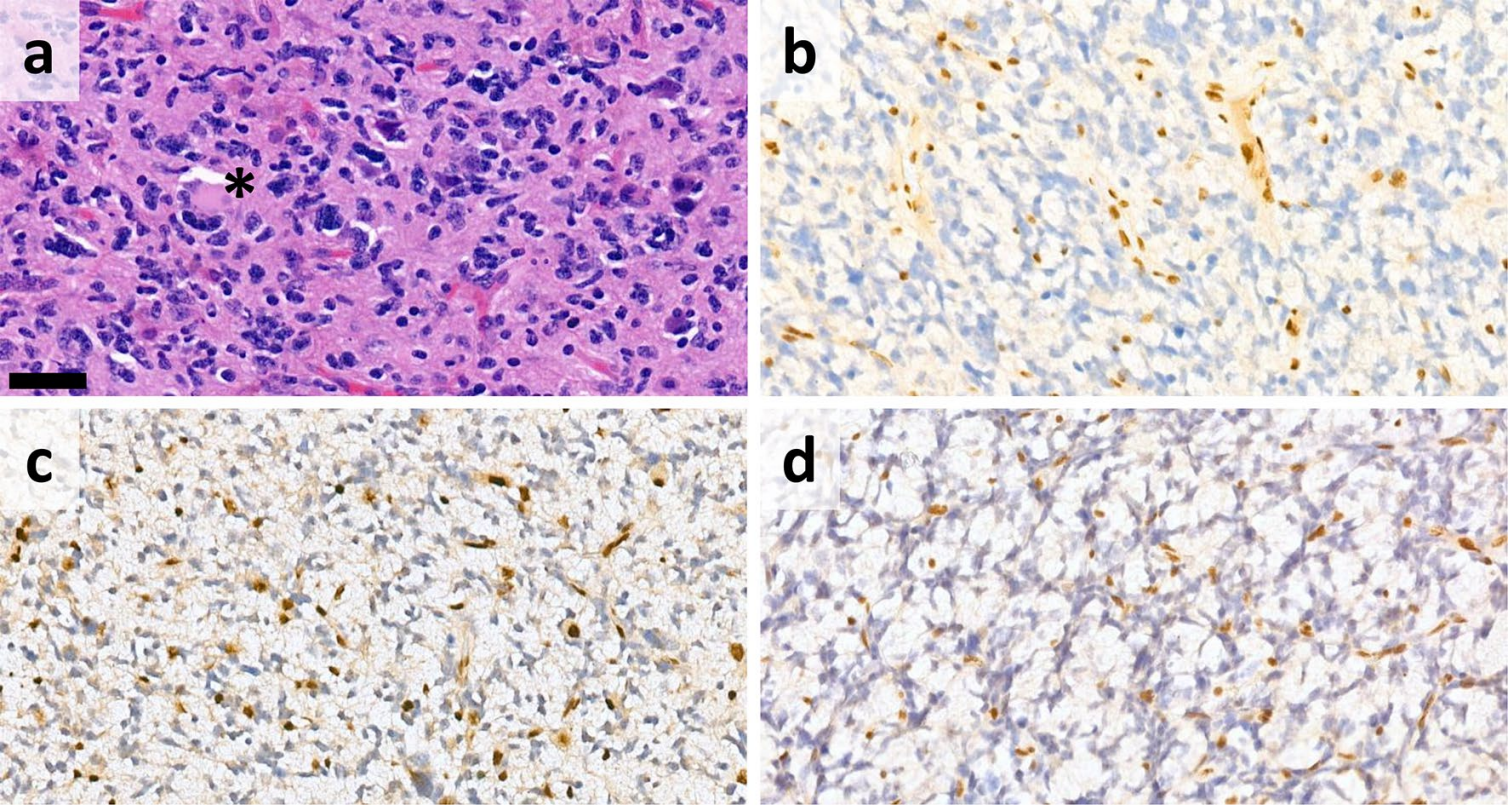
(**a**) H&E-stained sections from case #25 showed scattered multinucleated giant cells (black asterisk) (scale bar: 50 μm). Immunohistochemistry revealed loss of (**b**) ATRX, (**c**) MSH2 and (**d**) MSH6.

High TMB was detected exclusively in cases #17 and #25, both of which were ATRX-deficient. Microsatellite instability (MSI) analysis revealed MSI-high (MSI-H) status only in case #25 (Fig. 2). In case #17, CGP was performed on a recurrent GB. Both the primary and recurrent tumors showed oligodendroglioma-like morphology (Fig. 3a,b) as well as ATRX loss (Fig. 3c,d). In the recurrent tumor sample, a P mutation of the *MLH1* gene was found, accompanied by loss of both MLH1 and PMS2 immunoreactivities in the tumor cells (Fig. 3f,h). However, both MLH1 and PMS2 expressions were retained in the primary sample of the patient taken prior to radio-chemotherapy (Fig. 3e,g). A variant of uncertain significance (VUS) in the *MSH6* gene was also detected, but did not result in MSH6 loss. In addition to mismatch repair deficiency (MMR-d), this tumor harbored 2 LP mutations and 1 VUS in the *POLE* gene. Case #25 (Fig. 4a,b) harbored a VUS in the *MLH1* gene, but both MLH1 and PMS2 expressions were retained. In contrast, MSH2 and MSH6 expressions were lost in the tumor cells, without detectable genetic lesion in the encoding genes (Fig. 4c,d). VUSs in *MSH2*, *MSH6* and *MLH1* were also found in cases #15, #19 and #10, but without loss of the encoded proteins, high TMB or MSI-H (Fig. 2).

A conspicuously high number of HR repair-related genes harbored alterations (usually SNVs) in our cohort (Fig. 2). Among these, *PTEN* and *DNMT3A* were already described. Regarding others, P or LP alterations occurred in five ATRX-deficient tumors (*ARID1A* in case #23, *ARID2* in case #1, *ATM* in case #25, *CHEK2* in case #21 and *FANCA* in case #17), but only in one ATRX-intact case (*FANCI* in case #10).

For the detailed lists of variants fulfilling the inclusion criteria in each case, see Supplementary Table S2. Tabular summary of the variants, variant types as well as the frequency of affected cases are demonstrated on Supplementary Fig. S3.

## Discussion

In the study presented here, we analyzed the genetic patterns of ATRX-deficient IDH-wildtype and H3-wildtype adult high-grade gliomas in comparison with ATRX-intact GBs using CGP. To the best of our knowledge, this is the first complex genomic analysis specifically concentrating on this rare subgroup. In addition to confirming previous findings showing the mutual exclusivity of *ATRX* and *pTERT* mutations as well as the association of *NF1* mutations and ATRX loss, our study revealed novel, previously unreported genomic patterns with potential clinical significance. *PTEN* mutations were absent, while *DNMT3A* variants were enriched in ATRX-deficient tumors highlighting previously unknown genetic characteristics of this subgroup. In addition, MMR-d occurred in two peculiar ATRX-deficient tumors and the pattern of altered HR repair-related genes were substantially different in the examined cohorts.

ATRX deficiency and *pTERT* hotspot mutations were previously shown to be mutually exclusive^3^, which was clearly confirmed in our study. The main biological consequence of ATRX loss is the induction of ALT, which is the most important mechanism of telomere length maintenance in IDH-mutant and H3-altered diffuse astrocytic gliomas^12,13^. However, ATRX deficiency is not sufficient to induce ALT by itself, and other factors are also necessary. An example of these is the inhibition of the histone demethylase KDM4B in H3 G34R- and IDH-mutant astrocytic gliomas, as it was recently described^14^. In IDH- and H3-wildtype gliomas, the mechanisms of ALT are less well characterized. From this respect, considerable enrichment of *DNMT3A* mutations in the ATRX-deficient cases is particularly interesting. Each detected mutation has been described previously in various tumors (c.2259G>A in myelodysplastic syndrome^15^, c.2119G>A in GB^6^, c.1502A>G and c.1661G>A in acute myeloid leukemia^16,17^). *DNMT3A* mutations are well-known prognostic factors in acute myeloid leukemia^18^, but only sporadic occurrence has been reported in gliomas^6,19–21^. Considering previous observations showing ALT-related phenotypes in DNMT-deficient cells^22^, the cooperation of *ATRX* and *DNMT3A* mutations in telomere length maintenance can be suspected.

Among changes affecting RTK genes, *EGFR* amplification, a molecular diagnostic criterion of GB, was apparently, but not significantly less frequent in the ATRX-deficient group. This observation is not surprising, considering the association of *EGFR* amplification and *pTERT* mutations in GB^3^. Complex genetic events affecting the 4q12 region, containing the *PDGFRA* and *KIT* genes, were also detected in some cases. The peculiar complexity of genetic lesions in this region was previously observed by our group^23^ and others as well^24^, but these are not clearly associated with ATRX loss. Presence of fusions affecting RTK genes were relatively frequent in our cohort. The *SEPTIN14::EGFR*, *PTPRZ1::MET*^25^, *KDR::PDGFRA*^23^ and *FGFR3::TACC3*^11^ fusions are known from the literature, but the *PDGFRA::ADGRL3*, *WASL::MET* and *DNAJB6::MET* rearrangements involve previously unreported partner genes.

Among the members of PI3K-AKT pathway, *PTEN* is well known to harbor loss-of-function mutations in around 30–40% of GBs^26^. *PTEN* mutations occurred frequently in our ATRX-intact, but were absent in the ATRX-deficient tumors. According to the currently available data, *PTEN* mutations do not affect the prognosis of GB^27^, but may harbor potential predictive value. Clinical studies with immune checkpoint inhibitors produced generally disappointing results in primary and recurrent GB, but a minority of patients shows clinical response^28^. It was recently recognized that *PTEN* mutations are more frequent in recurrent GBs being resistant to PD-1 inhibitors^29^.

The association of *NF1* mutation and ATRX loss as well as ALT was previously described in high-grade diffuse gliomas and is accompanied by poor prognosis^30,31^. In our cohort, *NF1* mutations were more frequent in ATRX-deficient tumors, although the difference was not statistically significant. Other alterations related to the RAS–MAPK pathway including *KIAA1549::BRAF* fusion and *BRAF* p.V600E mutation occurred in two additional cases in the ATRX-deficient group. These data suggest that there is a trend towards RAS–MAPK pathway alterations in ATRX-deficient high-grade gliomas. According to recent observations, variants of RAS–MAPK pathway components, in particular, *BRAF* mutations, seem to be the most promising actionable targets in brain tumors^32^. Furthermore, abnormalities in this pathway are significantly enriched in GBs responding to immunotherapy^29^ and *NF1* mutation has been shown to induce an immunosuppressive, macrophage-microglia rich microenvironment in gliomas^33^. Inhibition of the RAS–MAPK pathway could serve as a valuable therapeutic tool by inducing sensitivity to immune checkpoint blockade in various tumors including breast cancer^34^. Our results regarding the frequency of *PTEN* and RAS–MAPK pathway alterations suggest that this approach may have a rationale in ATRX-deficient IDH-wildtype adult gliomas and ATRX loss could be a potential marker to predict responder cases to immunotherapeutic interventions.

MMR-d and subsequent genetic hypermutability were recently shown to be associated with at least partial giant cell morphology in GBs and are regarded to be important predictors of sensitivity to immune checkpoint inhibitors^20,35^. *NF1* mutations as well as *ATRX* and *DNMT3A* mutations were also shown to be significantly enriched in this GB variant suggesting a biological overlap with our group of interest. Tumors containing giant cells were remarkably, but not significantly enriched in our ATRX-deficient group, although only scattered giant cells were present in most of these cases. 6 of the 8 cases harboring *NF1* and/or *DNMT3A* mutations as well as 1 of 2 cases showing MMR-d (case #25) contained giant cells supporting the abovementioned previous observations, although the correlations were less evident in our cohort.

One of the ATRX-deficient samples with MMR-d (case #17) was a recurrent tumor showing oligodendroglioma-like morphology and harboring fusions involving the *NTRK2* and *LRRFIP2* genes. This rearrangement increases the peculiarity of this case, considering the rarity of fusions involving the *NTRK2* gene in adult high-grade gliomas^36^ and the previously unreported partner gene. An *LRRFIP2::ALK* fusion was recently described in a case of epithelioid fibrous histiocytoma^37^, but the exact mechanism by which *LRRFIP2* activates its partner genes remains to be elucidated. Among the detected *NTRK2*-fusions, the most likely to carry biological relevance is *LRRFIP2::NTRK2* involving the 3’ end of *NTRK2* with the protein kinase domain. It is worth noting, that the co-occurance of *NTRK2*-rearrangement and ATRX loss raises the possibility that this tumor represents the novel GTAKA category^8^. Loss-of-function mutation of *MLH1*, accompanied MLH1 and PMS2 loss as well as high TMB are likely to be related to therapy-induced hypermutation in this case, since both MLH1 and PMS2 expressions were intact in the primary tumor sample^38^. This tumor was classified as MSI-L, confirming previous observations regarding the low sensitivity of microsatellite instability as a marker of MMR-d in gliomas^38^. *POLE* mutations were also detected, neither of which affected the exonuclease domain^39^, but 2 of them (c.4773G>A; p.W1591* and c.2908G>A; p.G970S) were regarded to be LP. *POLE* variants were documented to be associated with constitutional MMR-d-related gliomas^40^ and the consecutive defect of DNA-polymerase proofreading might also contribute to the high TMB in this case.

The other ATRX-deficient, primary GB showing de novo high TMB and concomitant MSI-H harbored an *MLH1* mutation, which was classified as a VUS by our algorithm and was not accompanied by PMS2 loss. However, loss of MSH2 and MSH6 proteins was identified, without genetic alterations in their genes. Together with some similar previous observations^20,38^, this interesting finding raises the possibility of the existence of an epigenetic mechanism of MSH2 and MSH6 loss in gliomas, such as promoter hypermethylation^41^. Besides the already known constitutional and therapy-induced MMR gene defects, this is a possible new pathway of MMR gene inactivation in this group of tumors. Although results with immunotherapies in hypermutated gliomas were disappointing, potential utility of this approach still merits further investigation^38^.

The abovementioned connections between HR and ATRX deficiency are also intriguing from a therapeutic point of view. Recent preclinical data revealed an increased DNA-damage response and HR repair deficiency (HRD) in ATRX-deficient tumors, resulting in higher sensitivity to poly (ADP-ribose) polymerase (PARP)^42,43^ as well as ataxia telangiectasia and Rad3-related (ATR) inhibitors^43,44^. PARP inhibitors are emerging new therapeutic options in the treatment of primary and recurrent GB^45^, but further clinical evidence is necessary regarding their utility. The effects of these compounds are generally based on synthetic lethality in tumors showing HRD. The latter is rarely related to *BRCA1/2* mutations in high-grade gliomas, in contrast to breast or ovarian carcinomas. Instead, other genetic lesions producing a similar phenotype (“BRCAness”) include *IDH1/2* and *PTEN* mutations as well as EGFR and MYC overexpression^46^. According to our results, these changes are absent or rare in ATRX-deficient gliomas, but several mutations in other HR-related genes^47,48^ were found. Although the relevance of these non-*BRCA* gene mutations in the effects PARP inhibitors is not completely clarified, their synchronous presence with ATRX loss raises the possibility that ATRX-deficient high-grade gliomas are promising targets of PARP inhibitors.

According to recent clinical surveys, results of CGP have a low impact on the management of adult patients with high-grade gliomas^49^. Responses to molecularly targeted therapies are mostly related to well characterized driver mutations^32^. Detailed characterization of high-grade glioma subgroups is needed in order to facilitate future successful clinical trials and precisely predict responders. ATRX-deficient IDH-wildtype and H3-wildtype adult high-grade gliomas occur in small numbers even in large publicly available databases. Although somewhat limited by the low sample number, our study provides invaluable novel data regarding the unique genomic patterns of this subgroup being comparable to the histomorphological data as well as potential clinical significance. Our results suggest that these tumors may represent intriguing targets of immune checkpoint- and RAS–MAPK pathway inhibitor combinations as well as DNA repair inhibitors. Therefore, our findings could promote further studies towards more effective and personalized future therapies.

## Methods

### Tissue collection and clinical data

Formalin-fixed, paraffin-embedded (FFPE) tissue samples of the 25 included cases were obtained from the pathology departments of the following institutions: Semmelweis University (Department of Pathology and Experimental Cancer Research), County Hospital Győr, Petz Aladár Hospital and University of Szeged, from a 4-year-long period. At least two independent neuropathologists reviewed the hematoxylin and eosin (H&E) stained sections as well as available IHC and molecular data and confirmed the diagnosis according to the current WHO criteria^1^. All further molecular and IHC analyses were performed in the Department of Pathology and Experimental Cancer Research of the Semmelweis University. The IDH-wildtype and H3-wildtype status was confirmed by CGP in all cases (see below). Clinical data were obtained from the archives of the National Institute of Mental Health, Neurology and Neurosurgery and National eHealth Infrastructure.

### Immunohistochemistry (IHC)

IHC studies were performed according to the standard laboratory protocols on 3 μm thick sections, using a Leica Bond-Max automated immunostaining system (Leica Biosystems, Danvers, MA). For the reactions presented here, the following primary antibodies were used: ATRX (Sigma-Aldrich; polyclonal, HPA001906, 1:500), MLH1 (Cell Marque, G168-7289, 1:100), PMS2 (Leica Biosystems; NCL-L-PMS2, 1:100), MSH2 (Cell Marque, G219-1129, 1:100), MSH6 (Leica Biosystems; NCL-L-MSH6, 1:100).

### Comprehensive genomic profiling (CGP)

CGP was performed from FFPE samples using the Illumina TruSight Oncology 500 High-Throughput assay, then sequenced on an Illumina NextSeq 2000 NGS platform and finally analyzed with TruSight Oncology 500 Local App v2.1 as previously described^23^. Predetermined definition of TMB-high status was at least 10 mutations per megabase^50^. MSI status was determined by interrogation of 130 homopolymer MSI marker sites. MSI-H status was defined as ≥ 20%, MSI-low (MSI-L) as 20–1% and MS-stable (MSS) as < 1%, based on previous studies^51^.

Each alteration was classified according to the American College of Medical Genetics and Genomics and the Association for Molecular Pathology (ACMG-AMP) five-tier system^52^. Only P, LP changes as well as VUSs were included into this study. Synonymous SNVs as well as non-synonymous SNVs with variant allele frequency (VAF) below 2% and / or total depth lower than 100× were excluded from the analysis^32^. Regarding CNVs, low-level copy number gain was defined as copy numbers between 3 and 5, while amplifications as 5 or higher^53^. Fusions were designated according to the of HUGO Gene Nomenclature Committee (HGNC) recommendations^54^, thus the 5′ fusion partners were listed first (before the double colon). Potential germline variants were excluded based on a gnomAD frequency of 1%.

### *pTERT* mutation analysis

Difficulties regarding the detection of *pTERT* mutations are well known from the literature due to the GC-rich nature of this region^55^. Therefore, all cases were tested for *pTERT* hotspot variants (c.-124C>T and c.-146C>T) using Sanger sequencing on an ABI3500 platform (Thermo Fisher Scientific, Waltham, MA, USA) with custom-designed primers, namely forward: 5′-CACCCGTCCTGCCCCTTCACCTT-3′ and reverse: 5′-GGCTTCCCACGTGCGCAGCAGGA-3′.

### Statistical analysis

Kaplan–Meier analysis, Mann–Whitney test and Fisher’s exact tests were performed using GraphPad Prism software version 8.0 (San Diego, CA, USA). CGP revealed mostly scattered or balanced alterations in the analyzed groups, therefore, the statistical analysis was restricted to the associations of *pTERT*, *PTEN*, *DNMT3A* and *NF1* mutations as well as *EGFR* amplification with *ATRX* mutations. This was performed using Fisher’s exact tests followed by adjustment of *p*-values for multiple comparisons (*p*_adj_) using the Benjamini–Hochberg method. In all procedures, *p* or *p*_adj_ < 0.05 was regarded to be level of statistical significance. The detailed oncoplot presented on Supplementary Fig. S3 was created using Maftools v2.14 R-package^56^.

### Ethical approval and consent to participate

This retrospective study was conducted in accordance with the Declaration of Helsinki and informed consent was obtained from the participants. All procedures were approved by the National Scientific and Ethical Committee (TUKEB) of the Medical Research Council of Hungary (ETT) (statement: IV/51-1/2022/EKU).

### Supplementary Information

Supplementary Figures.

Supplementary Tables.

## Data Availability

The datasets used and/or analyzed during the current study are available from the corresponding author on reasonable request.

## Abbreviations

ALT: Alternative lengthening of telomeres
CGP: Comprehensive genomic profiling
CNV: Copy number variation
FFPE: Formalin-fixed, paraffin-embedded
FISH: Fluorescent in situ hybridization
GB: Glioblastoma
GTAKA: Glioneuronal tumor with ATRX alteration, kinase fusion and anaplastic features
H&E: Hematoxylin and eosin
HGAP: High-grade astrocytoma with piloid features
HR: Homologous recombination
HRD: Homologous recombination repair deficiency
IHC: Immunohistochemistry
InDel: Insertion and deletion
LP: Likely pathogenic
MMR-d: Mismatch repair deficiency
MSI: Microsatellite instability
NGS: Next-generation sequencing
OS: Overall survival
P: Pathogenic
RTK: Receptor tyrosine kinase
SNV: Single nucleotide variant
TMB: Tumor mutational burden
VAF: Variant allele frequency
VUS: Variant of uncertain significance
WHO: World Health Organization
