# Supplementary material for: Novel, clinically relevant genomic patterns identified by comprehensive genomic profiling in ATRX-deficient IDH-wildtype adult high-grade gliomas
Source: Sci Rep. 2023 Oct 27;13:18436. doi: 10.1038/s41598-023-45786-w (PMC10611758; doi:10.1038/s41598-023-45786-w)

Supplementary figures

Novel, clinically relevant genomic patterns identified by comprehensive genomic profiling in ATRX-deficient IDH-wildtype adult high-grade gliomas

Gábor Bedics, Péter Szőke, Bence Bátai, Tibor Nagy, Gergő Papp, Noémi Kránitz, Hajnalka Rajnai, Lilla Reiniger, Csaba Bödör, Bálint Scheich

Figure S1. (**a**) Comparison of age distribution (mean ± standard deviation) and (**b**) Kaplan-Meier curves showing the percent overall survival depending on time elapsed after surgery of ATRX-deficient and ATRX-intact cases.

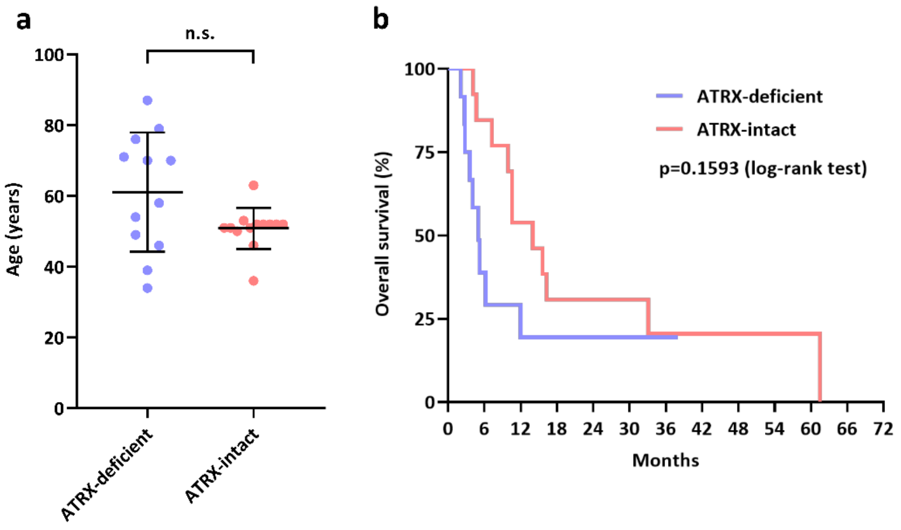

Figure S2. (**a**) Circos plot illustrating the fusions detected in our cohort (the plot was generated using CIRCOS). (**b**) Morphological features of case #2 with pilocytic astrocytoma-like features, including the presence of numerous Rosenthal fibers (black arrowhead) (scale bar: 50 µm). The insert shows the result of FISH analysis demonstrating the presence of *KIAA1549::BRAF* fusion in a tumor cell (probe colors: red: *BRAF*, green: *KIAA1549*, blue: *HIPK2*). (**c**) Representative image demonstrating the morphology of case #16 harboring *FGFR3::TACC3* fusion according to CGP.

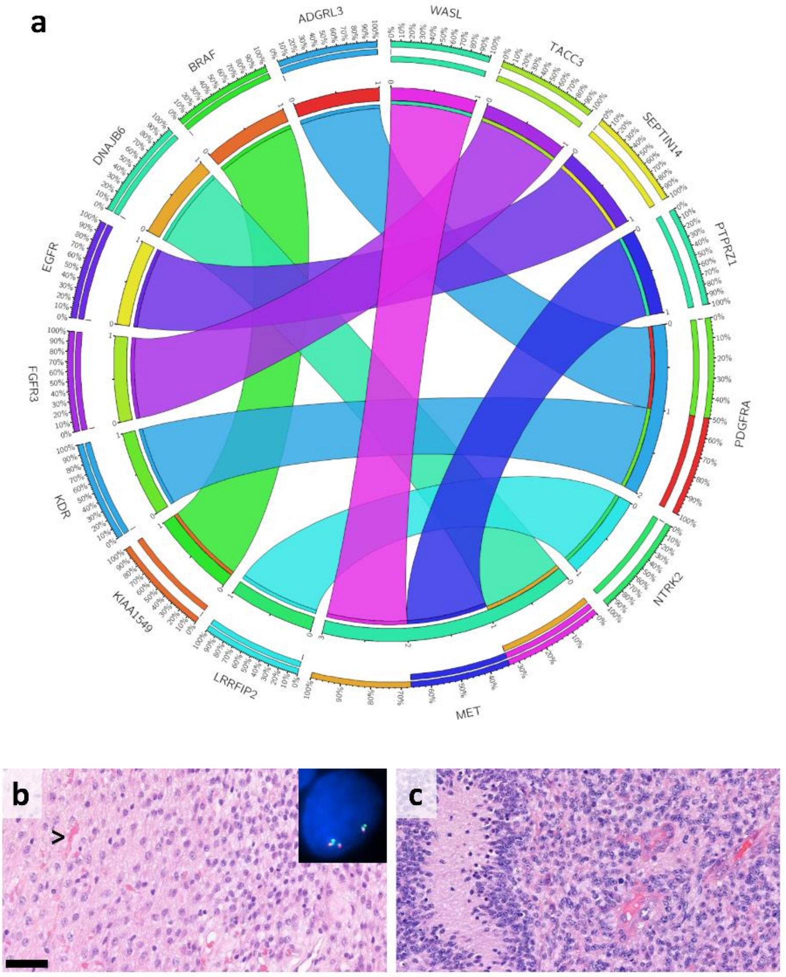

Figure S3. Detailed summary of all detected variants in the included ATRX-deficient and ATRX-intact gliomas using CGP (the plot was created using Maftools v.2.14).

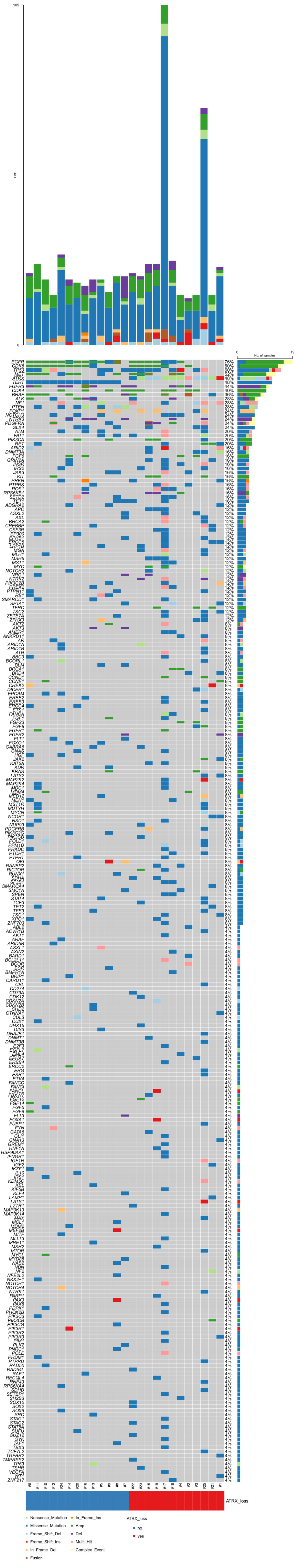

Supplement: Supplementary file 1 — Supplementary Figures. [file 41598_2023_45786_MOESM1_ESM.pdf]
